# Dosimetric Therapeutics as Applied to Pneumonia and Bronchitis

**Published:** 1888-01

**Authors:** 


### Selections.

### *DOSIMETRIC THERAPEUTICS AS APPLIED TO PNEUMONIA AND BRONCHITIS.*

The system of therapeutics, as taught by Dr. Ad. Burggraeve, by which he claims to arrest acute maladies by the prompt exhibition of alkaloids in minute doses, at frequent intervals, is among the recent innovations in the field of medicine. The "jugulation of acute diseases," as Dr. Burggraeve styles his system, is on trial in all countries, and many eminent practitioners report the new system of dosimetry, as it is termed, productive of good results. The essential fevers in particular are benefited and abridged by dosimetric methods; diphtheria is more successfully combated; and pulmonary affections of acute type rapidly relieved. These are a few of the results claimed by Dr. Burggraeve and his disciples.

To illustrate the subject, I extract from the *Dosimetric Medical Review* two cases—one of pneumonia, the other of acute bronchitis—as reported by Dr. Ferran. Although the first case was treated before the preparation of the dosimetric alkaloidal granules by ch. Chanteaud & Co., of Paris, still it will be seen that the treatment is practically the same, and that the granules could have been substituted, with less trouble and more accuracy, for the solutions used by Dr. Ferran. The foundation of treatment in acute pulmonary attacks is pilocarpine, as employed by Dr. Ferran. Case II. better illustrates dosimetric therapeutics than Case I, which is mainly to show the applicability of dosimetry to pneumonia, as well as to bronchial inflammations.

### Double Pneumonia (Complete).

Case I.—On a night of the month of March, 1878, I had just placed myself at table, when an adjutant of the Sixth Artillery, Mr. Irleman, entered my house much perturbed. "My son has just arrived from the Lyceum," he said, "and he is going to have the croup, for he can no longer draw his breath, and is suffocating." I went to the child, who was thirteen or fourteen years of age, and I found, not an affection of the larynx, but a double, complete pneumonia; so complete was it, indeed, that the respiration came only in a light wave of air from the inferior part of the right lung; the little patient was in reality commencing to suffocate.

I had never seen so generalized a case of pneumonia, and I had no doubt however that, with the habitual medications, this case would have been promptly mortal. I did not fail to instruct the father as to the gravity of the situation.

Nevertheless, a certain peculiarity which I had often observed came to my mind, viz., that in the treatment of pneumonia, the respiration is freer and the general condition more reassuring while the patient is perspiring. Consequently, I thought of having recourse to the infusion of *pilocarpus jaborandi*, for at this epoch its alkaloid, pilocarpine, was scarcely known, and did not exist in pharmacy. For a greater security, I did not care to use the commercial leaves, so often valueless, and employed the jaborandi pills of one of the importers.

A first dose (five grammes) was administered at once in three parts in infusion, one cupful at a time; then, a second in eight and a-half hours, followed by a third in ten hours. The habitual muco-salivary sputa, as also the transpiration, was enormous. But, after ten o'clock at night, respiration was ample, and after eleven I could easily see that my patient was saved.

On the morning of the next day, there was a fluxionary return, and in the evening a return of the fever; but the congestion occurred only in isolated points, and was not a serious matter. The continuation of the transpiration treatment, together with tinct. aconite in minute doses with revellents, arrested the fever and the congestion within three days—a result which would have been reached still more promptly with the use of the defervescent granules.

Without doubt, the effects of rapid decongestion obtained on this occasion with jaborandi may be attributed to the fact that the congestion was recent. But these effects are not the less marvelous, and are wholly beyond comparison with those obtained with the ordinary medicines.

Since that time, I have had occasion to employ jaborandi, or the nitrate of pilocarpine in granules, on several occasions in partial pneumonia, and their prompt efficaciousness has always been manifested.

The effects of the nitrate and the chlorhydrate of pilocarpine are much less irritating upon the stomach than those of jaborandi, and are always sure, while those of the leaves of the plant are uncertain, and the salts are also infinitely more convenient for administration, particularly for children. It follows, therefore, that pilocarpine should be substituted wherever possible for jaborandi.

When we wish to obtain from pilocarpine its sudatory effects, the average dose for an adult should be twenty-five granules, administered in two portions with an interval of from two to five minutes. It is indispensable to give them fasting, otherwise they incite painful vomiting. But the effects of sudation are always preceded by a very abundant mucus and salivary discharge, which makes itself felt upon the larynx and the bronchial passages. It also happens at times that the medicament incites contractions at the neck of the bladder. For this reason, it is well to cause patients to urinate before taking it.

But the anti-congestive action of pilocarpine is not confined to pneumonia; it is exercised in no less remarkable a manner in laryngitis in bronchitis of whatever nature they may be. I could cite very many examples of this, but as they all resemble each other, I describe only such cases as are most interesting from a practical point of view.

### Infantile Bronchitis.

Case II.—In the bronchial affections of children at the breast, the nitrate of pilocarpine in granules is a precious medicament, not only on account of the attenuated doses in which it may be administered, but from the fact that it is tasteless. Striking examples of the advantageous use of the medicament in such cases, are shown in the following notes:

At L.'s house, civil engineer; child five months old; heavy attack of bronchitis during the night. On January 17th (morning), I made my first visit; a looch of white oxide antimony upon the night table told me that a gentleman of the Old School had preceded me. The medicament had been prescribed by a confrere, but no one could prevail upon the child to take it. Thanks to Chanteaud's dosimetric granules, my treatment was much better carried out, and it was in other respects an improvement. At nine o'clock in the morning, ten minutes after my visit, the child took a first granule, which was perfectly well retained. Then half a granule was given every half hour and, later on, every hour, after having swaddled the child in warm flannel. At the end of three hours of this treatment, amelioration was manifested by a diminution of the cough; and toward two P. M., the relatives, who up to that time had been very anxious, made the joyful discovery that the child took with avidity the breast-milk it had refused on the previous evening. During the same night, he was able to sleep in his cradle—

which he had not done for two days—and by the middle of the following day, everything went on as usual.

[The writer gives another case in a child of seven months, in which the treatment was equally prompt and efficacious, the patient being cured in a very few hours.—Ed. D. M. R.]

I may cite another case, a child of thirteen months (though not yet weaned), who had been suffering for three days from bronchitis, and had a very intense fever. At my visit on the sixth of the month I, found by auscultation an obstruction of all the superior part of the right lung, which had become impermeable to respiration, and there were faint rales at the inferior part. The cough was frequent and intense; the nares dilated at each inspiration, and the horizontal position was impossible.

Prescription: The child to be swaddled in linens and flannels, and in an outer covering of worsted; four granules of pilocarpine; one at once, then a half granule every half hour (in solution). When transpiration was well established, four granules of aconitine to be given, one-half a granule at a dose. In the night, two granules of pilocarpine, two of aconitine and one of brucine to be taken in four doses, *i. e.*, from hour to hour.

During the same night, the child became more tranquil, and respired more freely.

On the 7th and 8th, the same treatment was continued in decreasing, doses, according as the degree of progress toward amelioration became visible. On the morning of the 9th, this amelioration seemed to me so far advanced, that I discontinued my visits. Since this time, the child has enjoyed good health.

I limit to the foregoing my citations of the cases of pneumonia and bronchitis cured rapidly by the use of Chanteaud's granules of pilocarpine, aconitine, etc. A large number of similar cases would only fatigue the attention of the reader, while it could not add to the value of these citations as applied to every-day practice. I believe I have said enough to attract the attention of all who seek only for the truth in good faith and with unprejudiced minds. As for others, what I write does not concern them, and these I shall never make any effort to convince. They will finally be obliged, however, to give way to the evidence of facts; the public will have something to say about that matter.

